# Severe somatoform and dysautonomic syndromes after HPV vaccination: case series and review of literature

**DOI:** 10.1007/s12026-016-8820-z

**Published:** 2016-08-09

**Authors:** Beniamino Palmieri, Dimitri Poddighe, Maria Vadalà, Carmen Laurino, Carla Carnovale, Emilio Clementi

**Affiliations:** 1 Department of General Surgery and Surgical Specialties, University of Modena and Reggio Emilia Medical School, Surgical Clinic, Modena, Italy; 2 Department of Paediatrics, ASST Melegnano e Martesana, Vizzolo Predabissi, MI Italy; 3 Unit of Clinical Pharmacology, Department of Biomedical and Clinical Sciences, National Research Council-Institute of Neuroscience, University Hospital L. Sacco, University of Milano, Milan, Italy

**Keywords:** HPV, Vaccine, ADR, Symptoms, Adjuvant, Autoimmune syndrome, ASIA

## Abstract

Human papilloma virus (HPV) is recognized as a major cause for cervical cancer among women worldwide. Two HPV vaccines are currently available: Gardasil^®^ and Cervarix^®^. Both vaccines enclose viral antigenic proteins, but differ as to the biological systems of culture and the adjuvant components. Recently, a collection of symptoms, indicating nervous system dysfunction, has been described after HPV vaccination. We retrospectively described a case series including 18 girls (aged 12–24 years) referred to our “Second Opinion Medical Network” for the evaluation of “neuropathy with autonomic dysfunction” after HPV vaccination. All girls complained of long-lasting and invalidating somatoform symptoms (including asthenia, headache, cognitive dysfunctions, myalgia, sinus tachycardia and skin rashes) that have developed 1–5 days (*n* = 11), 5–15 days (*n* = 5) and 15–20 days (*n* = 2) after the vaccination. These cases can be included in the recently described immune dysfunction named autoimmune/inflammatory syndrome induced by adjuvants (ASIA). HPV vaccine, through its adjuvant component, is speculated to induce an abnormal activation of the immune system, involving glia cells in the nervous system too. Further researches should aim at defining the pathological and clinical aspects of these post-vaccination diseases and identifying a genetic background predisposing to these adverse reactions.

## Introduction

Human papilloma virus (HPV) infection has been recognized to have a significant role in the occurrence and progression of several tumours, such as cervical cancer, representing one of the most common malignancies among women worldwide [1].

Following this discovery, some vaccines have been produced to prevent such a sexually transmitted infection, against the most frequently involved serotypes, namely HPV-6, HPV-11, HPV-16 and HPV-18. Particularly, the latter two have been estimated to account for 70 % of cases of cervical cancer [2].

Currently, two HPV vaccines are available on the market: Gardasil^®^ (a quadrivalent vaccine covering serotypes 6, 11, 16 and 18) was licensed in 2006 and Cervarix^®^ (a bivalent vaccine against HPV-16 and HPV-18) in 2009. Both vaccines enclose self-assembled antigenic pentamers of L1 proteins, produced by DNA recombinant technology, but differ as to the biological system of culture and the adjuvant components, as summarized in Table 1. Gardasil^®^ is produced by *Saccharomyces cerevisiae* cells, and viral antigens are pooled with the adjuvant aluminium hydroxyphosphate sulphate (225 μg). Cervarix^®^ is obtained through a L1-recombinant baculovirus-infected insect (*Spodoptera frugiperda* Sf9, *Trichoplusia ni* Hi-5), and the adjuvant component is represented by ASO4 complex that is made of aluminium hydroxide and *Salmonella minnesota* lipopolysaccharide extract. The intramuscular (IM) administration protocol encompasses three bimonthly injections that elicit a strong persisting antibody titre against HPV serotypes over six months and a good control of its related infection [3–5].

**Table 1:** Characteristics of human papillomavirus (HPV) vaccines

Type of HPV vaccine	Gardasil^®^	Cervarix^®^
Protection against HPV genotypes (amount)	Quadrivalent human papillomavirus (types 6, 11, 16, 18) recombinant vaccine	Bivalent human papillomavirus (types 16, 18) recombinant vaccine
Vaccine composition	Each dose contains 20 µg of HPV 6 L1 protein, 40 µg of HPV 11 L1 protein, 40 µg of HPV 16 L1 protein, and 20 µg of HPV 18 L1 protein	Each dose contains 20 µg of HPV 16 L1 protein and 20 µg of HPV 18 L1 protein
Producer cells	*Saccharomyces cerevisiae*	*Spodoptera frugiperda* SF9, *Trichoplusia ni* Hi 5
Adjuvants and inactive ingredients	Each dose of the vaccine contains 225 µg of aluminium [as amorphous aluminium hydroxyphosphate sulphate (AAHS) adjuvant], 9.56 mg of sodium chloride, 0.78 mg of l-histidine, 50 µg of polysorbate 80, 35 µg of sodium borate and water for injection	Each dose of the vaccine contains 500 µg of aluminium (hydroxide salt), 50 μg of 3-O-desacyl-4′ monophosphoryl lipid A, 4.4 mg of sodium chloride, 0.624 mg of sodium dihydrogen phosphate dihydrate and water for injection
Route of administration	Intramuscular injection	Intramuscular injection
Dosage	9–26 years: 0.5 mL dose at 0, 2 and 6 months	9–14 years: two 0.5 mL doses each of 0.5 ml. The second dose given between 5 and 13 months after the first dose From 15 years and above: three 0.5 mL doses at 0, 1, 6 months
Manufacturing	Merck Canada Inc.	GlaxoSmithKline—GSK

So far, more than 100 million HPV vaccine doses have been administered worldwide and it has been shown to provide protective and long-lasting antibody titres against the virus. The largest study investigating the safety of HPV vaccine included almost 300,000 young girls administered with nearly 700,000 doses of the quadrivalent vaccine: no increases in the number of cases of autoimmune and/or neurological diseases were detected. Moreover, a pooled analysis of five clinical trials involving 11,778 quadrivalent HPV vaccinated people and 9686 placebo-treated controls, aged 9–26 years, showed that 0.2 % of both vaccine and placebo recipients had dropped out because of adverse drug reactions (ADRs). Most dropouts occurred after the first dose and the most severe cases were due to autoimmunity; however, those events affected equally the vaccine and placebo groups [6–18].

The temporal relationship between the vaccination and the development of immune-mediated diseases raised the issue of the possible aetiological or co-causative role of such an immunization procedure, which may occur in patients with a genetic predisposition. Moreover, long-standing neurological and/or psychiatric symptoms after HPV vaccine being refractory to any treatment elicited further concerns [19–21].

Recently, these ADRs have been pooled in a heterogeneous group of medical conditions (including also Gulf War syndrome, macrophagic myofasciitis, siliconosis, sick building syndrome) named autoimmune/inflammatory syndrome induced by adjuvants (ASIA). The common feature of different ASIA clinical conditions is considered to be the previous exposure to an external stimulus (including vaccines) triggering an undefined immune-mediated response elicited by its adjuvant properties [22–25].

Here an incidental finding of a small cluster of young females with severe and persistent neurological and/or psychiatric symptoms arising after HPV vaccination led us to review critically this issue.

## Case series

In 2015, through our “Second Opinion Medical Network”, we have been asked for evaluating a girl with cervical lymphadenitis, headache, severe persistent fibromyalgia-like pain, paresthaesia, chronic fatigue, insomnia, cognitive impairment, abnormal menstrual cycles and fingers/toes vascular abnormalities (changes in skin colour and temperature perception). Such a clinical picture was defined (by the neurologists evaluating the patient) as “neuropathy with autonomic dysfunction”. Later, some other families from Northern and Central Italy, whose daughters had been complaining of similar symptoms after HPV vaccination, were referred to our centre through the mother of this first patient. That fact allowed us to visit and analyse a case series including 18 young girls aged 12–24 years who developed post-vaccination phenomena after receiving Gardasil^®^ (*n* = 9) or Cervarix^®^ (*n* = 9). Patients’ and immunization features are reported in Table 2. An overview of the clinical manifestations considering the temporal association with HPV immunization is reported in Table 3. Current and persisting clinical problems are summarized in Table 4.

**Table 2:** Patients’ and immunization features

Patients (N)	18	
Age range	12–24 years	Mean age 15.2 years
Weight range	44–87 kg	Mean weight 56.3 kg
Height range	150–174 cm	Mean height 162.3 cm

Childhood diseases	Chickenpox (varicella)	MMR (measles, mumps and rubella) vaccine	Mononucleosis	Scarlet fever	Poliomyelitis vaccine	Fifth disease	Cytomegalovirus (CMV) infection
	18 (patients)	8 (patients)	4 (patients)	2 (patients)	2 (patients)	1 (patient)	1 (patient)
HPV vaccine type	Gardasil^®^			Cervarix^®^			
	9 (patients)			9 (patients)			
Year and age patients that started HPV vaccination	Gardasil^®^			Cervarix^®^			
	2008–2015 (range)			2008–2015 (range)			
	11 years (mean age)			12 years (mean age)			
No. doses	One dose		Two doses		Three doses		
	4 (patients)		8 (patients)		6 (patients)

**Table 3:** Description of ADRs related to HPV immunization

	After first dose	After second dose	After third dose
**Symptoms (within 5 days post-vaccination)**			
*Injection site reactions*			
Pain	2	2	1
Uncontrollable and involuntary movement of the limb	1		
Swelling	1	1	
*Systemic reactions*			
Low-grade fever	3	5	1
Headache	5	5	2
Recurrent syncope	4	3	
Persistent convulsive hunger	1		
Irritability	1	2	
Epileptic seizures		1	
Temporary speech loss		1	
Lower limbs paraesthesia and paresis		1	
Hot flushes		1	
Severe stomach pain	2	1	1
Insomnia	3	2	
Hypersensitivity reactions	1		
Leg muscle pain (myalgia)	1	3	1
Gait and orthostatic posture impairment		1	
Excessive sweating	1		
Vomiting	1		
**Symptoms (5–15 days post-vaccination)**			
*Systemic reactions*			
Asthenia	2	5	1
Persistent thirst		3	
Severe hands and feet itching	1		
Optic neuritis	1		
**Symptoms (15–20 days post-vaccination)**			
*Systemic reactions*			
Amenorrhoea		1	
Skin rashes	1	1	
Tachycardia		1	1
Difficult breathing		1	
Weight loss		1

**Table 4:** Current and persisting ADRs in the case series

Current symptoms	Patients (n.)	Duration of symptoms (years)
Dizziness	6	4
Headache	12	9
Fever	3	4
Skin rashes	1	1
Asthenia	9	4
Myalgia	13	5
Sinus tachycardia	2	7
Vascular skin abnormalities	13	5
Abdominal pain	4	3
Confusion	3	2
Memory loss	6	3
Concentration problems	11	4
Depression	3	2
Disordered sleep	8	3
Candidiasis	1	2
Nausea	1	3
Paresthaesia	1	3

## Results

Eighteen girls (aged 12–24 years, current mean age being 15.2 years) have been included in this case series. As concerns their past personal history, all of them reported chickenpox during childhood, and some had mononucleosis (*n* = 4), scarlet fever (*n* = 2), fifth disease (*n* = 2) or CMV-related eruption (*n* = 1). During childhood, eight patients received the MMR (measles, mumps and rubella) vaccine, in addition to the regular immunization schedule administered at that time, including at least DTP, poliomyelitis and HBV. Nine girls were vaccinated against HPV via the quadrivalent vaccine (Gardasil^®^), between January 2008 and December 2015, and nine girls received HPV-16/18 vaccine (Cervarix^®^) between January 2008 and December 2015 (Cervarix^®^). The mean age at HPV immunization was 11 and 12 years (Table 2).

The ADRs described in Table 3 occurred after the first IM injection in four girls, after the second dose in eight patients and after the third boost in six of them. As expected, the first two groups refused the following doses of the HPV immunization protocol. Based upon the time interval between vaccination and symptoms onset, we divided our patients in three groups (Table 3): (1) girls whose symptoms appeared 1–5 days post-vaccination (*n* = 11), (2) girls whose symptoms appeared 5–15 days after the vaccination (*n* = 5) and (3) girls whose symptoms occurred 15–20 days post-vaccination (*n* = 2).

Local ADRs were also reported: pain at the injection site (*n* = 5), involuntary movement at the injection arm, especially hand tremor (*n* = 1), swelling (*n* = 2).

The systemic reactions observed 1–5 days after the vaccination consisted in low-grade fever (*n* = 9), headache (*n* = 12), recurrent syncope (*n* = 7), persistent convulsive hunger (*n* = 1), irritability (*n* = 3), epileptic seizures (*n* = 1), transient speech loss (*n* = 1), lower limbs paresthaesia and paresis (*n* = 1), hot flushes (*n* = 1), severe stomach pain (*n* = 4), insomnia (*n* = 5), hypersensitivity reactions (e.g. itchy skin rash, rhinitis) (*n* = 1), muscle pain (*n* = 5), severe gait impairment and orthostatic intolerance (*n* = 1), excessive sweating (*n* = 1), vomiting (*n* = 1).

Clinical manifestations occurring 5–15 days after vaccination were asthenia (*n* = 8), excessive thirst (*n* = 3), severe hands and feet itching (*n* = 1), optic neuritis (*n* = 1). Finally, other patients complained of symptoms after 15–20 days after vaccination: amenorrhoea (*n* = 1), skin rashes (*n* = 2), tachycardia (*n* = 2), difficult breathing (*n* = 1), weight loss (*n* = 3).

Among the most common and persistent symptoms (Table 4), the patients complained of memory and concentration impairment (*n* = 17), muscle pain (*n* = 13), finger and toes vascular abnormalities with skin colour and temperature changes (*n* = 13), headache (*n* = 12), asthenia (*n* = 9), dizziness (*n* = 6), abdominal pain (*n* = 4), low-grade fever (*n* = 3), sinus tachycardia (*n* = 2), skin rashes (*n* = 1).

## Discussion

Immunization practice definitely modified the epidemiology of human infectious diseases since its introduction some centuries ago: indeed, a dramatic reduction of the morbidity and/or mortality in the populations recruited by mass vaccine campaigns was obtained [26, 27]. Despite these positive achievements, concerns on safety emerged from a number of clinical reports describing post-vaccination polymorphous phenomena [23, 28].

However, available studies reported no statistical association between vaccines (including those against HPV) and the incidence of autoimmune diseases, except for a few and well-defined situations [29, 30]. In the past, for instance, Guillain–Barrè syndrome was causally associated with the swine influenza vaccination campaign of 1976–1977. Currently, idiopathic thrombocytopenic purpura (ITP) has been related to vaccination against MMR, but the wild and natural viral infections resulted to have a tenfold greater risk to develop ITP, which supported the vaccination practice anyway. Similar observations have been made for other immune-mediated diseases following several vaccinations. Thus, the available evidence suggested that vaccines should not be considered a risk factor leading to a greater occurrence of well-defined immune-mediated and neurological diseases, compared to the natural infections that vaccines aim at preventing [23, 31, 32].

Moreover, both the use of standardized protocols and the coordinated surveillance by the regulatory drug agencies have currently erased vaccine-related diseases due to malpractice (e.g. infected materials, incomplete inactivation of infectious agents, gross errors of administration). Obviously, despite the best medical practice, vaccines as any other drug, can display unforeseen side effects: these intrinsic risks are relatively rare and are considered acceptable in front of the health benefits produced in terms of life quality and expectancy. Moreover, the warnings to avoid vaccinations in people with allergies to some components, with history of previous ADRs and with transient (e.g. during pregnancy) or permanent (e.g. severe primary and secondary immunodeficiency) impairment of the immune system, substantially reduced the incidence of complications [33]. Unfortunately, unpredicted ADRs still occur and the individual genetic background has been supposed to be responsible for these phenomena, including autoimmune diseases and/or neurological/psychiatric syndromes, without a specific or identifiable pathologic alteration. Recently, some authors reasoned that all these heterogeneous post-vaccination phenomena might be the consequence of some immune dysfunction, putatively activated by the adjuvant rather than by antigenic vaccine fractions. Thus, they named these medical conditions as ASIA [34, 35].

The adjuvant is devoted to enhance the immune response to the sensitizing molecules (antigens or haptens); yet they may contain some pathogen-associated molecular patterns (PAMPs) and/or create a mild tissue injury exposing some damage-associated molecular patterns (DAMPs), which are recognized by the innate immune system through specific pattern recognition receptors (PRRs) [21, 36–38].

If autoimmune or immune-mediated phenomena were related to a vaccination, the cross-reactive adaptive immune response to both antigenic and adjuvant components of vaccine might be considered. Therefore, at the site of injection, at afferent lymph nodes or also at distant organs, autoreactive immune processes may be activated by the vaccine adjuvant and not only by the antigenic part of the vaccine. The central and peripheral nervous system has been supposed to be one of the targets of this autoimmune reaction, leading to neurological and/or psychiatric symptoms, diagnosed as being ASIA, in some (genetically) predisposed individuals [37–40].

Our case series included a wide spectrum of post-vaccination general, neurological and psychiatric symptoms during and/or early after the scheduled HPV immunization. Most patients showed an “acute phase” characterized by a variable combination of clinical manifestations, including low-grade fever, skin rashes, muscle pain, headache and sensorial disturbances. Its onset occurred at a variable time after HPV immunization, ranging from hours to days. Then, this acute phase subsided in some weeks, but the girls developed some chronic and/or recurrent symptoms resembling chronic fatigue, fibromyalgia or other functional somatic syndromes. The clinical pictures displayed by each patient were actually polymorphous, except for asthenia, concentration problems, sensorial impairment (related to temperature perception, especially), recurrent or persisting muscle pain, paresis and headache. Previously, all these girls declared excellent fitness and wellness, including sport practice. No significant co-morbidities or previous relevant diseases were reported before the HPV immunization.

Currently, the diagnosis of ASIA is based upon the criteria proposed by Shoenfeld [39, 41], as reported in Table 5. It can be diagnosed whether two major criteria or one major and two minor criteria have been ascertained. In our case series, one of the major criteria is satisfied by definition, as all patients are joined by the exposure to HPV vaccine, as an external stimulus endowed of adjuvant properties. As summarized in Table 6, all patients presented at least one more of the major criteria and/or two minor criteria, making a diagnosis of ASIA be consistent.

**Table 5:** Proposed diagnostic criteria of autoimmune/inflammatory syndrome induced by adjuvants (ASIA)

**Major criteria** *Previous exposure to an external stimulus* (i.e. vaccine, adjuvant, silicone, nucleic acids, fragments of bacterial cell walls) *One of the following “typical” manifestations:* Myalgia/myositis, muscle weakness Arthralgia and/or arthritis Chronic fatigue, non-refreshing sleep or sleep disturbances Neurological manifestations (especially if associated with demyelination) Memory loss and cognitive impairment Fever Dry mouth *Improvement of symptoms after the removal of the triggering agent* *Typical biopsy of the involved organs*
**Minor criteria** *Appearance of autoantibodies or autoantibodies directed against the suspected adjuvant* *Other clinical manifestations* (i.e. functional somatic syndromes) *Association with specific HLA haplotypes* (i.e. HLA-DRB1, HLA-DQB1) *Development of autoimmune diseases*
**Diagnostic requirements:** Two major criteria One major criteria + two minor criteria

**Table 6:** ASIA diagnostic criteria in our case series

Patients	Major criteria (1st)	Major criteria (2nd)	Minor criteria (1st)	Minor criteria (2nd)
# 1	Cervarix^®^	Typical symptoms (CFS, myalgia, arthralgia)	Autoantibodies (ANA, anti-cardiolipin IgM)	Other clinical manifestations (fibromyalgia)
# 2	Cervarix^®^	Typical symptoms (low-grade fever, memory loss, CFS)	–	–
# 3	Gardasil^®^	Typical symptoms (muscle weakness, arthralgia, CFS)	–	Other clinical manifestations (neuropathy)
# 4	Gardasil^®^	Typical symptoms (muscle weakness, dry mouth, sleep disturbances, CFS)	–	
# 5	Gardasil^®^	Typical symptoms (low-grade fever, skin rashes, cognitive impairment)	HLA haplotype (HLA-DRB1)	Other clinical manifestations (fibromyalgia)
# 6	Cervarix^®^	Typical symptoms (muscle weakness, skin rashes, sleep disturbances, memory loss, CFS)	–	Development of autoimmune disease (diabetes mellitus type 1)
# 7	Gardasil^®^	Typical symptoms (muscle weakness, recurrent syncope, pins and needles)	–	–
# 8	Gardasil^®^	Typical symptoms (muscle weakness, dry mouth	Autoantibodies (anti-thyroglobulin and anti-TPO)	Development of autoimmune disease (diabetes mellitus type 1)
# 9	Gardasil^®^	Typical symptoms (muscle weakness, skin rashes, cognitive impairment, CFS)	–	Other clinical manifestations (fibromyalgia)
# 10	Cervarix^®^	Typical symptoms (muscle weakness, CFS)	–	Development of autoimmune diseases (Optic neuritis)
#11	Cervarix^®^	Typical symptoms (arthralgia, cognitive impairment, muscle weakness)	–	Other clinical manifestations (fibromyalgia)
# 12	Cervarix^®^	Typical symptoms (arthralgia, muscle weakness, dizziness, recurrent syncope, asthenia)	–	–
# 13	Gardasil^®^	Typical symptoms (nausea, asthenia, insomnia, syncope)	–	Other clinical manifestations (fibromyalgia, Raynoud syndrome)
# 14	Gardasil^®^	Typical symptoms (arthralgia, sleep disturbances)	Autoantibodies (anti-TG and anti-TPO)	Development of autoimmune disease (autoimmune thyroiditis)
# 15	Cervarix^®^	Typical symptoms (arthralgia, skin rashes)	–	Development of autoimmune disease (Schonlein–Henoch purpura and IgA nephropathy)
# 16	Gardasil^®^	Typical symptoms (fever, myalgia, myositis, cognitive impairment)	–	Other clinical manifestations (fibromyalgia)
# 17	Cervarix^®^	Typical symptoms (fever, CFS, sleep disturbances)	–	Other clinical manifestations (irritable bowel disease, fibromyalgia)
# 18	Cervarix^®^	Typical symptoms (arthralgia, myalgia, CFS)	Autoantibodies (anti-cardiolipin IgM and IgG, anti-2GPI IgM and IgG)	Other clinical manifestations (pseudo-neurological somatoform disorder)

The description of our case series aimed at analysing clinical phenomena, being putatively immune mediated and arising after HPV immunization. The latency of the onset of symptoms is quite variable, but it can be consistent with the occurrence of cell-mediated and/or antibody response against some neurological/neuromuscular structures.

The majority of post-vaccination ADRs were consistent with severe fibromyalgia and chronic pain syndrome, according to the literature criteria.

Because of these symptoms, at least 10 of these eighteen young girls developed a long-standing social impairment (school absence, sport suspension and daily activity impairment).

Certainly, the diagnostic criteria for ASIA need to be improved and this medical category requires a better clinical, immunological and, possibly, genetic definition. Indeed, in addition to the aforementioned diagnostic criteria, the diagnosis is currently supported by a strict temporal association with the onset of symptoms and, of course, by the exclusion of other well-defined infectious, immune-mediated and oncologic diseases. ASIA is thought to represent an infrequent side effect of vaccinations due to an adjuvant or adjuvant-like stimulus acting on a predisposed, but not well-defined, genetic background [39]. Because of its rarity, its heterogeneous clinical expression and the frequent absence of objective pathological lesions and immunological signatures, ASIA might have escaped to pre-clinical and post-marketing safety studies on vaccines. Indeed, the definition of ASIA as post-vaccination event originated by a study by Zafrir and co-workers on patients affected with Gulf War syndrome who had received HBV immunization [42].

Among commercialized vaccines, those against HPV have received a lot of descriptions of medical conditions being consistent with the diagnosis of ASIA, regardless the type of vaccine. Recently, Pellegrino et al. carried out an interesting analysis of HPV vaccine-related ASIA epidemiology in the vaccine adverse event reporting system (VAERS), which is a large database collecting almost 30,000 post-vaccination ADRs per year. Pooling the data until the end of 2013, the authors reported more than 26,000 ADRs after HPV vaccine: of these, about 8 % have been labelled as being probable or possible ASIA, according to the Shoenfeld and Agmon-Levin’s guideline for the diagnosis. A strong causality evaluation between HPV vaccination and ASIA has been supposed in this study [43]. Unfortunately, most descriptions of HPV post-vaccination phenomena consistent with ASIA have been single cases and small case series, where only the temporal link was evident.

A recent observational analysis promoted by UK Medicines and Healthcare products Regulatory Agency (MHRA) investigated the association between bivalent HPV vaccine and chronic fatigue syndrome (CFS) that is considered one of the main expressions or evolution of ASIA. No increase of CFS incidence was noticed in girls aged 12–20 years old after the introduction of such an immunization [44].

Moreover, in 2015, the European Medicine Agency (EMA) revised the safety profile of HPV vaccines as regards the occurrence of complex regional pain syndrome (CRPS) and postural orthostatic tachycardia syndrome (POTS). Such a revision was carried out through the Pharmacovigilance Risk Assessment Committee (PRAC) and the Global Advisory Committee on Vaccine Safety (GACVS), which were not able to find enough scientific evidence so far to support a clear causal link between HPV vaccine and the aforementioned syndromes and, thus, to alter the current recommendations for HPV immunization programme [45].

These studies were prompted by the burden of reports regarding syncope, asthenia and pain syndromes occurring after HPV immunization [46, 47]. Recently, Martinez-Lavin published an interesting clinical report where two girls have developed severe incapacitating fibromyalgia-like illness, after one and two doses of HPV inoculation, respectively [48].

This same author speculated that post-vaccination fibromyalgia, POTS and CRPS might be due to a dysautonomia, precisely a sympathetic nervous system dysfunction, because of a small fibre neuropathy triggered by the HPV immunization in susceptible people. Actually, such a hypothesis has been endorsed by Kinoshita et al. [49], who described a case series of 44 girls (aged 11–17 years) complaining of several symptoms after HPV immunization. Interestingly, in addition to headache, general fatigue, limb pain and coldness, most patients showed signs of vascular instability at the extremities, including a slight to moderate decrease of toes and fingers temperature, as many patients of our case series did.

The aforementioned EMA investigation had been prompted mainly by a retrospective analysis (carried out in Denmark) on 53 patients developing orthostatic intolerance and other symptoms consistent with autonomic dysfunction within two months after the quadrivalent HPV vaccination [50]. The authors stated a recognizable pattern of somatoform symptoms (headache, localized or diffuse neuropathic pain, dysautonomic disturbances, excessive fatigue and cognitive dysfunctions), where different clinical entities, namely POTS, CRPS, fibromyalgia and CFS, could be included. ASIA might be the diagnostic category including all these syndromes, but the inclusion criteria should be substantiated through further studies providing new insights into its neuropathic pathophysiology and adjuvant-triggered/immune-mediated pathogenesis. In this perspective, the first and mandatory step could be focusing the epidemiology of these post-vaccination phenomena and selecting/collecting an appropriate study population of girls complaining of this pattern of symptoms. Martinez-Lavin et al. recently published the results of questionnaires proposed to 45 patients developing chronic disturbances within 3 months after HPV vaccination, by means of three validated self-applied questionnaires evaluating the different groups of symptoms described after HPV immunization or, more in general, in ASIA patients: (1) the 2010 ACR Fibromyalgia Diagnostic Criteria Survey; (2) the COMPASS-31 test, exploring the autonomic dysfunction; (3) the S-LANSS questionnaire, evaluating the neuropathic component in pain syndromes [51]. We suggest drawing a specific questionnaire summarizing all these clinical aspects, to be administered to all the subjects receiving HPV vaccination in a perspective observational study. Such an approach could provide certain data on the real incidence of ASIA after HPV immunization, definitely ruling out the potential bias deriving from pre-existing subjective complaints. Then, a standardized and international consensus protocol of investigation ought to be planned on these identified cohorts of patients, in order to clarify the existence and the mechanisms of this puzzling syndrome.

Although no cause/effect evidence between HPV immunization and non-organic symptoms has been detected so far, the strong burden of concerns expressed by the parents’ associations from several European countries should not be underscored at all.

A database of 35 young girls immunized against HPV through Gardasil^®^ between 2008 and 2013 in France, who showed similar symptoms as our case series (and who received also a final diagnosis of autoimmune disease in some cases), was published by an independent parents’ association [52].

In Spain, the Asociacion de Afectadas pro la Vacuna del Papiloma (AAVP) collected 42 cases from 2008 to 2014: in addition to few cases of demyelinating/autoimmune diseases, most patients developed somatoform/functional disorders or not well-defined patterns of symptoms without a final diagnosis [53].

Again, in the UK, another parents’ association, named Association of HPV Vaccine Injured Daughters (AHVID), currently counts 265 members: through a questionnaire which has been responded to by almost 100 families so far, variable patterns of neuropathic disorders, being labelled as POTS, CRPS, CFS or fibromyalgia, were reported in most cases [54].

All these alerting reports are not institutional and cannot be reputed as systematic epidemiologic investigations; however, in our opinion, they have to be taken into adequate account to reach definite conclusions upon the relationship between HPV vaccines and ASIA/functional somatoform diseases, through further studies. This will be the only way to clarify the real risk of HPV vaccines, as regards these specific health issues: that will help to retract all potential and/or unfounded concerns related to HPV immunization, and, thus, will reinforce the perception of safety and effectiveness of HPV vaccines among people all over the world. Conversely, the identification of a genetically and/or immunologically cohort of young girls being potentially exposed to these kinds of diseases might lead to the selective exclusion from this immunization practice.

Indeed, like the UK Medicines and Healthcare products Regulatory Agency (MHRA) and other European Pharmacovigilance, other systems have been notified about this kind of side effects regarding HPV vaccines. For instance, the Netherland Pharmacovigilance Centre Lareb received 1271 reports of Adverse Events Following Immunization (AEFI) with Cervarix^®^ since the introduction of the National Immunization Programme in 2009, according to the data analysis updated at December 2015: 231 reports were about long-lasting AEFI, whose duration was 2 months or more, and most related complaints were persistent/recurrent fatigue, headache, dizziness, musculoskeletal discomfort and syncope [55]. Interestingly, after consulting the available medical records and the healthcare practitioners dealing with these patients, no medical explanation for all these long-lasting AEFI was found. According to this analysis, a causal relation between Cervarix^®^ and the reported long-lasting AEFI could not be concluded, but neither excluded, based upon the available data. Previously, in 2013, the same institution edited an overview of the reports on long-lasting fatigue following immunization with Cervarix^®^. As many others, the authors observed that the age of vaccinated girls had to be taken into account as potential confounding factor, but they concluded that additional epidemiological research was needed anyway, in order to confirm or reject a causal link [56]. It is well known that severe and/or long-lasting fatigue, like other somatoform symptoms, are not uncommon in young females, but this issue could be ascertained through an appropriate epidemiological and observational investigation. The girls affected by these symptoms after HPV immunization in our case series, as well as in databases reported by parents’ associations, had been completely healthy and had never complained of fatigue or somatoform symptoms before the administration of one or more doses of the HPV vaccine.

A plan of prospective and specific epidemiological studies, among active surveillance of post-vaccination ADRs, addressed to face the HPV immunization major concerns, was attempted in Italy through the National Centre of Epidemiology, Surveillance and Health Promotion. The second report on post-marketing surveillance of vaccines in Italy described the side effects in a cohort of 12,990 female subject aged 9–16 years who received free immunization through the Local Health System Institutions in the period going from June 2009 to December 2011. The main complaints observed were local reactions and symptoms like headache, muscles and joint pains. However, this surveillance was limited to symptoms arising within 15 days after the vaccination. Moreover, this analysis included only girls who voluntarily accepted to participate to the study, moreover, it was not possible to ascertain the effective surveillance coverage in each recruited vaccination centre [57]. It might be reasonable comparing girls receiving and refusing freely available HPV vaccination, planning a longer follow-up period and collecting data by telephone interviews taking advantage of specific questionnaires for self-reported symptoms. This approach will assess the actual burden of long-lasting AEFI and might identify a specific population to be investigated in order to detect genetic traits and/or prognostic biological markers, predicting an increased risk for long-lasting AEFI. Currently, few studies describing a positive correlation between post-vaccination phenomena and genetic variants, such HLA haplotypes or single nucleotide polymorphisms, are currently available and have been mainly addressed to transient symptoms, such as fever, arthralgia and other systemic ADRs [19, 58]. Recently, an increased risk of narcolepsy, which could be included in the spectrum of ASIA, was described after AS03-adjuvanted H1N1 vaccination and, importantly, the authors proposed an association with HLA-DQB1*06:02 [59, 60]. Thus, the possibility to find similar genetic or biological markers might increase the safety of immunization programs and decrease the existence of vaccine refusal movements.

Conclusively, our study highlighted some important post-vaccination phenomena temporally linked to HPV immunization, which needs further epidemiological analysis and biological investigations in order to establish or exclude a causal relation.

The exact incidence of these post-vaccination syndromes in the general population has not been known yet and a definite cause/effect link between HPV vaccine-related immune activation and symptoms onset has to be established. However, the studies on fibromyalgia, chronic pain with dysautonomia and CFS considered that a previous subclinical and spontaneous reaction to viral agents could be often involved in the start of pain and functional impairment. Therefore, a link between HPV vaccination and some neuromuscular and systemic impairment might be possible, considering also the immunization properties displayed by HPV vaccines, according to several studies [61].

Moreover, the HPV vaccine formula, containing also high polysorbate 80 (50 mcg) concentration, might also induce a greater meningeal permeability leading to a facilitated entrance of many substances to the central nervous system. Based upon these observations, it might be speculated that this vaccine—and not others—could induce some abnormal activation of immune competent cells in the central nervous system, such as the glia [62, 63]. Finally, we believe that our commitment should be planning epidemiological and genetic investigations in order to clarify the existence and pathophysiology of HPV-related syndromes. Hopefully, this approach might lead to a screen test for this risk and, eventually, to prevent it: indeed, these syndromes are currently an orphan drug area, as all the experimented therapies have not shown any significant beneficial effect. Last but not least, a proper treatment for these somatoform syndromes is urgently needed, as the current painkilling drugs, including opiates, resulted to be ineffective, or paradoxically, pain enhancers.

## References

[1] Shiller JT, Lowy DR (2014). Virus infections and human cancer: an overview. Recent Results Cancer Res.

[2] Malik H, Khan FH, Ahsan H (2014). Human papillomavirus: current status and issues of vaccination. Arch Virol.

[3] Schiller JT, Lowy DR, Markowitz LE. Vaccine immunology. In: Plotkin SA, Orenstein WA, Offit PA, editors. Vaccines. 6th ed. Philadelphia: Saunders; 2013. p. 235–56.

[4] O’Hagan DT. Preparation methods and research protocols. In: Vaccine adjuvants. Humana Press; 2000. p. 1–342.

[5] Bryan JT (2007). Developing an HPV vaccine to prevent cervical cancer and genital warts. Vaccine.

[6] Arnheim-Dahlstrom L, Pasternak B, Svanstrom H, Sparen P, Hviid A (2013). Autoimmune, neurological, and venous thromboembolic adverse events after immunisation of adolescent girls with quadrivalent human papillomavirus vaccine in Denmark and Sweden: cohort study. BMJ.

[7] Nicol AF, de Andrade CV, Russomano FB, Rodrigues LS, Oliveira NS, Provance DW, Nuovo GJ (2015). HPV vaccines: their pathology-based discovery, benefits, and adverse effects. Ann Diagn Pathol.

[8] Stratton K, Ford A, Rusch E, Clayton EW. Adverse effects of vaccines, evidence and causality. The National Academies Press; 2012. p. 1–866. 24624471

[9] Gatto M, Agmon-Levin N, Soriano A, Manna R, Maoz-Segal R, Kivity S, Doria A, Shoenfeld Y (2013). Human papillomavirus vaccine and systemic lupus erythematosus. Clin Rheumatol.

[10] Hariri S, Bennett NM, Niccolai LM, Schafer S, Park IU, Bloch KC (2015). Reduction in HPV 16/18-associated high grade cervical lesions following HPV vaccine introduction in the United States—2008–2012. Vaccine.

[11] Paavonen J, Jenkins D, Bosch FX, Naud P, Salmerón J, Wheeler CM (2007). Efficacy of a prophylactic adjuvanted bivalent L1 virus-like-particle vaccine against infection with human papillomavirus types 16 and 18 in young women: an interim analysis of a phase III double-blind, randomised controlled trial. Lancet.

[12] Joura EA, Leodolter S, Hernandez-Avila M, Wheeler CM, Perez G, Koutsky LA (2007). Efficacy of a quadrivalent prophylactic human papillomavirus (types 6, 11, 16, and 18) L1 virus-like-particle vaccine against high-grade vulval and vaginal lesions: a combined analysis of three randomised clinical trials. Lancet.

[13] Centers for Disease Control and Prevention (2013). (CDC) National and state vaccination coverage among adolescents aged 13–17 years–United States, 2012. MMWR Morb Mortal Wkly Rep.

[14] Tabrizi SN, Brotherton JM, Kaldor JM, Skinner SR, Cummins E, Liu B (2012). Fall in human papillomavirus prevalence following a national vaccination program. J Infect Dis.

[15] Hutchinson DJ, Klein KC (2008). Human papillomavirus disease and vaccines. Am J Health Syst Pharm.

[16] Petrosky E, Bocchini JA, Hariri S, Chesson H, Curtis CR, Saraiya M (2015). Use of 9-valent human papillomavirus (HPV) vaccine: updated HPV vaccination recommendations of the advisory committee on immunization practices. MMWR Morb Mortal Wkly Rep.

[17] Garland SM, Hernandez-Avila M, Wheeler CM, Perez G, Harper DM, Leodolter S (2007). Quadrivalent vaccine against human papillomavirus to prevent anogenital diseases. N Engl J Med.

[18] McCormack PL (2014). Quadrivalent human papillomavirus (types 6, 11, 16, 18) recombinant vaccine (gardasil): a review of its use in the prevention of premalignant anogenital lesions, cervical and anal cancers, and genital warts. Drugs.

[19] Pellegrino P, Falvella FS, Perrone V, Carnovale C, Brusadelli T, Pozzi M (2015). The first steps towards the era of personalised vaccinology: predicting adverse reactions. Pharmacogenomics J.

[20] Pellegrino P, Carnovale C, Pozzi M, Antoniazzi S, Perrone V, Salvati D (2014). On the relationship between human papilloma virus vaccine and autoimmune diseases. Autoimmun Rev.

[21] Pellegrino P, Clementi E, Radice S (2015). On vaccine’s adjuvants and autoimmunity: current evidence and future perspectives. Autoimmun Rev.

[22] Shoenfeld Y, Agmon-Levin N (2011). ASIA—Autoimmune/inflammatory syndrome induced by adjuvants. J Autoimm.

[23] Poddighe D, Castelli L, Marseglia GL, Bruni P (2014). A sudden onset of a pseudo-neurological syndrome after HPV-16/18 AS04-adjuvated vaccine: might it be an autoimmune/inflammatory syndrome induced by adjuvants (ASIA) presenting as a somatoform disorder?. Immunol Res.

[24] Stubgen JP (2014). A review on the association between inflammatory myopathies and vaccination. Autoimmun Rev.

[25] Karussis D, Petrou P (2014). The spectrum of post-vaccination inflammatory CNS demyelinating syndromes. Autoimmun Rev.

[26] Plotkin SL, Plotkin SA. A short history of vaccination. In: Plotkin SA, Orenstein WA, Offit PA, editors. Vaccines. 6th ed. Philadelphia: Saunders; 2013. p. 1–13.

[27] Fine P (2014). Science and society: vaccines and public health. Public Health.

[28] Van der Laan JW, Gould S, Tanir JY (2015). Safety of vaccine adjuvants: focus on autoimmunity. Vaccine.

[29] Wraith DC, Goldman M, Lambert PH (2003). Vaccination and autoimmune disease: what is the evidence. Lancet.

[30] Grimaldi-Bensouda L, Guillemot D, Godeau B, Bénichou J, Lebrun-Frenay C, Papeix C (2014). Autoimmune disorders and quadrivalent human papillomavirus vaccination of young female subjects. J Intern Med.

[31] Offit PA, De Stefano F. Vaccine safety. In: Plotkin SA, Orenstein WA, Offit PA, editors. Vaccines. 6th ed. Philadelphia: Saunders; 2013. p. 1464–80.

[32] Rajantie J, Zeller B, Treutiger I, Rosthöj S (2007). Vaccination associated thrombocytopenic purpura in children. Vaccine.

[33] Tozzi A (2004). Field evaluation of vaccine safety. Vaccine.

[34] Guimarães LE, Baker B, Perricone C, Shoenfeld Y (2015). Vaccines, adjuvants and autoimmunity. Pharmacol Res.

[35] Soriano A, Nesher G, Shoenfeld Y (2015). Predicting post-vaccination autoimmunity: who might be at risk?. Pharmacol Res.

[36] Pulendran B, Ahmed R (2011). Immunological mechanisms of vaccination. Nat Immunol.

[37] Kuroda E, Coban C, Ishii KJ (2013). Particulate adjuvants and innate immunity: past achievements, present findings and future prospects. Int Rev Immunol.

[38] Mohan T, Verma P, Rao DN (2013). Novel adjuvants & delivery vehicles for vaccines development: a road ahead. Indian J Med Res.

[39] Perricone C, Colafrancesco S, Mazor RD, Soriano A, Agmon-Levin N, Shoenfeld Y (2013). Autoimmune/autoinflammatory syndrome induced by adjuvants (ASIA) 2013: unveiling the pathogenic, clinical and diagnostic aspects. J Autoimm..

[40] Cruz-Tapias P, Agmon-Levin N, Israeli E, Anaya JM, Shoenfeld Y (2013). Autoimmune (auto-inflammatory) Syndrome Induced by Adjuvants (ASIA)—Animal models as proof of concept. Curr Med Chem.

[41] Shoenfeld Y. Video Q&A: what is ASIA? An interview with Yehuda Shoenfeld. BMC Med. 2013;11:118. 10.1186/1741-7015-11-118 PMC3662178 23635355

[42] Zafrir Y, Agmon-Levin N, Shilton T, Shoenfeld Y (2012). Autoimmunity following hepatitis B vaccine as part of the spectrum of Autoimmune(Auto-inflammatory) Syndrome Induced by Adjuvants (ASIA): analysis of 93 cases. Lupus..

[43] Pellegrino P, Perrone V, Pozzi M, Carnovale C, Perrotta C, Clementi E, Radice S (2015). The epidemiological profile of ASIA syndrome after HPV vaccination: an evaluation based on the Vaccine Adverse Event Reporting Systems. Immunol Res.

[44] Donegan K, Beau-Lejdstrom R, King B, Seabroke S, Thomson A, Bryan P (2013). Bivalent human papillomavirus vaccine and the risk of fatigue syndromes in girls in the UK. Vaccine.

[45] European Medicine Agency (EMA): Pharmacovigilance Risk Assessment Committee (PRAC). Assessment report EMA/762033/2015:Human papillomavirus (HPV) vaccines. Pharmacovigilance Risk Assessment Committee (PRAC). http://www.ema.eu/docs/en_GB/document_library/Referrals_document/HPV_vaccines_20/Opinion_provided_by_Committee_for_Medicinal_Products_for_Human_Use/WC5000197129.pdf. Accessed 19 Mar 2016.

[46] Blitshteyn S (2014). Postural tachycardia syndrome following human papillomavirus vaccination. Eur J Neurol.

[47] Martinez-Lavin M (2014). Fibromyalgia-like illness in 2 girls after human papillomavirus vaccination. J Clin Rheumatol.

[48] Martínez-Lavín M (2015). Hypothesis: Human papillomavirus vaccination syndrome—small fiber neuropathy and dysautonomia could be its underlying pathogenesis. Clin Rheumatol.

[49] Kinoshita T, Abe RT, Hineno A, Tsunekawa K, Ikeda S (2014). Peripheral sympathetic nerve dysfunction in adolescent Japanese girls following immunization with the human papillomavirus vaccine. Intern Med.

[50] Brinth L, Theibel AC, Pors K, Mehlsen J (2015). Suspected side effects to the quadrivalent human papilloma vaccine. Dan Med J..

[51] Martínez-Lavín M, Martínez-Martínez LA, Reyes-Loyola P (2015). HPV vaccination syndrome. A questionnaire-based study. Clin Rheumatol.

[52] List des effects indesirables suite a la vaccination HPV en France. Confidential document received by the corresponding author.

[53] Asociacion de afectadas por la vacuna del papiloma (AAVP). Spanish AAVP database of HPV vaccine affected girls. Confidential document received by the corresponding author.

[54] Association of HPV Vaccine Injured Daughters (AHVID). Database of HPV vaccine affected girls. Confidential document received by the corresponding author.

[55] The Netherlands Pharmacovigilance Centre Lareb. Long-lasting adverse events following immunization with Cervarix. 2015:1–21. http://lareb.nl/Signalen/Lareb_rapport_HPV_dec15_03. Accessed 19 Mar 2016.

[56] The Netherlands Pharmacovigilance Centre Lareb. Overview of reports of long-lasting fatigue following immunisation with Cervarix^®^. 2013:1–4. http://lareb.nl/Signalen/KWB_2013_3_cerva.aspx. Accessed 19 Mar 2016.

[57] Santuccio C, Trotta F, Felicetti P, Da Cas R, Menniti-Ippolito F, Raschetti R et al. Rapporto sulla sorveglianza postmarketing dei vaccini in Italia 2011:1–52. http://www.agenziafarmaco.gov.it/sites/default/files/Copertina%20+%20Rapporto%20vaccini%202011_0.pdf. Accessed 19 Mar 2016.

[58] Mitchell LA (1998). HLA-DR class II associations with rubella vaccine-induced joint manifestations. J Infect Dis.

[59] Ahmed SS, Schur PH, MacDonald NE, Steinman L (2014). Narcolepsy, 2009 A(H1N1) pandemic influenza, and pandemic influenza vaccinations: what is known and unknown about the neurological disorder, the role for autoimmunity, and vaccine adjuvants. J Autoimmun.

[60] Partinen M, Kornum BR, Plazzi G, Jennum P, Julkunen I, Vaarala O (2014). Narcolepsy as an autoimmune disease: the role of H1N1 infection and vaccination. Lancet Neurol.

[61] Faust H, Toft L, Sehr P, Müller M, Bonde J, Forslund O (2016). Human Papillomavirus neutralizing and cross-reactive antibodies induced in HIV-positive subjects after vaccination with quadrivalent and bivalent HPV vaccines. Vaccine.

[62] Göppert TM, Müller RH (2005). Polysorbate-stabilized solid lipid nanoparticles as colloidal carriers for intravenous targeting of drugs to the brain: comparison of plasma protein adsorption patterns. J Drug Target.

[63] Masserini M (2013). Nanoparticles for brain drug delivery. ISRN Biochem.

